# Radiographically occult bone marrow metastasis in relapsed small cell lung cancer presenting with rapidly progressive thrombocytopenia: a case report

**DOI:** 10.3389/fonc.2026.1926902

**Published:** 2026-08-26

**Authors:** Jingli Li, Fang Liu, Shijia Yu, Meng He, Jian Sun, Zhe Chen, Ying Qian, E Qin

**Affiliations:** 1 Department of Pulmonary and Critical Care Medicine, Shaoxing People’s Hospital, Shaoxing, Zhejiang, China; 2 School of Medicine, Shaoxing University, Shaoxing, Zhejiang, China; 3 Department of Pathology, Shaoxing People’s Hospital, Shaoxing, Zhejiang, China; 4 Department of Hematology, Shaoxing People’s Hospital, Shaoxing, Zhejiang, China; 5 Department of Clinical Laboratory Center, Shaoxing People’s Hospital, Shaoxing, Zhejiang, China

**Keywords:** bone marrow metastasis, case report, next-generation sequencing, small cell lung cancer, thrombocytopenia

## Abstract

**Background:**

Small cell lung cancer (SCLC) is an aggressive neuroendocrine malignancy that frequently relapses after initial chemoradiotherapy. Bone marrow involvement in SCLC has been reported previously, but relapse confined predominantly to the marrow may be difficult to recognize when skeletal imaging remains negative. We report a diagnostically discordant presentation of relapsed SCLC characterized by rapidly progressive thrombocytopenia, marked biochemical progression without corresponding radiographic evidence of skeletal disease, and pathological confirmation of bone marrow metastasis supported by immunophenotypic and molecular findings.

**Case description:**

A 76-year-old Chinese man was diagnosed with limited-stage right upper-lobe SCLC in August 2025. Initial positron emission tomography/computed tomography showed a right upper-lobe lesion with mediastinal and right hilar lymph node involvement, without definite distant or skeletal metastasis; brain magnetic resonance imaging showed no intracranial metastasis. He received six cycles of etoposide plus carboplatin followed by thoracic radiotherapy to the right lung and mediastinum. Approximately 3 months after radiotherapy, he was admitted with fatigue and dyspnea. No antitumor therapy was administered between May 19 and June 6, 2026. During the final hospitalization, the platelet count decreased rapidly from 88×10^9/L to 18×10^9/L, accompanied by anemia, nucleated red blood cells, elevated lactate dehydrogenase and ferritin, and markedly increased tumor markers, including neuron-specific enolase, pro-gastrin-releasing peptide, carbohydrate antigen 125, and carbohydrate antigen 19-9. Chest and abdominal imaging and bone scintigraphy did not reveal definite bone metastasis. Bone marrow aspiration and biopsy performed on June 1 confirmed metastatic SCLC. Immunohistochemistry showed epithelial and neuroendocrine differentiation, mutant-type p53 expression, and loss of RB. Flow cytometry detected CD45-negative abnormal epithelial cells expressing CD326, CD56, and CD117. Targeted next-generation sequencing of the residual bone marrow specimen identified TP53 and RB1 alterations. Despite supportive treatment, the patient deteriorated rapidly and died on June 6, 2026.

**Conclusions:**

This case highlights a clinically aggressive but radiographically occult pattern of SCLC relapse in which rapidly progressive cytopenias and biochemical progression preceded detectable skeletal abnormalities. Negative bone scintigraphy and the absence of destructive bone lesions should not delay bone marrow examination when the clinical and laboratory findings suggest marrow infiltration.

## 1 Introduction

Small cell lung cancer (SCLC) accounts for approximately 15% of all lung cancers and is characterized by rapid proliferation, early dissemination, and initial sensitivity to platinum-based chemotherapy and radiotherapy (1). Despite favorable initial responses, most patients ultimately experience relapse. Although immune checkpoint inhibitors, consolidation durvalumab after chemoradiotherapy for limited-stage disease, and newer agents such as lurbinectedin and DLL3-directed tarlatamab have expanded treatment options, durable disease control after relapse remains difficult (2–4). The liver, brain, adrenal glands, bone, and distant lymph nodes are common metastatic sites (5). Median overall survival generally ranges from 16 to 24 months in limited-stage disease and from 6 to 12 months in extensive-stage disease, although outcomes vary with treatment era and patient selection (6). Bone marrow involvement is less commonly recognized in routine clinical practice, but it can be clinically devastating because it may lead to progressive thrombocytopenia, anemia, leukoerythroblastosis, coagulation abnormalities, bleeding, infection, and rapid multiorgan failure (7, 8).

The diagnosis of bone marrow metastasis in SCLC can be difficult, particularly when cytopenia occurs in a patient who has previously received cytotoxic therapy. Thrombocytopenia and anemia may be attributed to prior chemotherapy, infection, drug-related marrow suppression, immune-mediated cytopenia, bleeding, or consumptive coagulopathy (9, 10). In addition, bone marrow infiltration may not be accompanied by overt cortical bone destruction or positive bone scintigraphy, especially when tumor cells predominantly colonize the marrow compartment rather than producing radiographically apparent osseous lesions (11). Therefore, progressive or unexplained cytopenia in patients with relapsed SCLC should prompt consideration of early bone marrow evaluation, even when conventional imaging does not reveal definite bone metastasis.

Genomic profiling can provide complementary biological information in selected cases of SCLC (12). Concurrent inactivation of TP53 and RB1 is a canonical molecular feature of the disease (13), although alterations detected by tumor-only sequencing of bone marrow specimens require cautious interpretation because of potential contributions from clonal hematopoiesis. Bone marrow involvement in SCLC has been reported previously (4), and the present case does not establish this metastatic site as a novel manifestation of the disease. Rather, it documents a diagnostically discordant pattern of relapse in which rapidly progressive thrombocytopenia and marked biochemical progression occurred despite negative bone scintigraphy and the absence of destructive skeletal lesions. Bone marrow morphology and biopsy established the diagnosis, while immunohistochemistry, flow cytometry and targeted sequencing provided complementary phenotypic and molecular support. This case therefore illustrates when bone marrow evaluation may be warranted despite an absence of radiographic evidence of skeletal metastasis. We present this article in accordance with the CARE reporting checklist.

## 2 Case presentation

**Figure 1:**
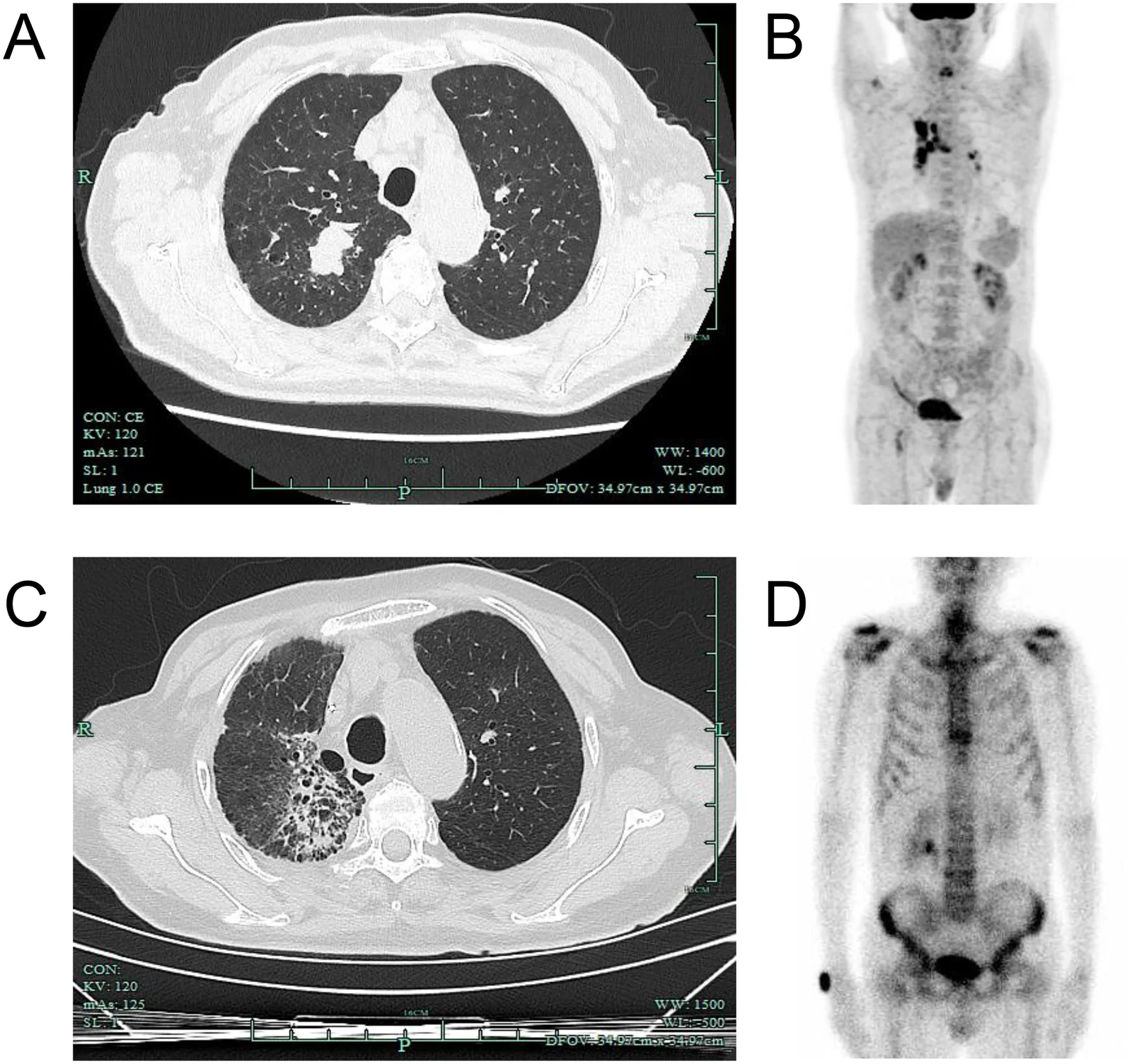
Radiological findings at initial diagnosis and terminal admission. **(A)** Initial contrast-enhanced chest CT showed a right upper-lobe lesion. **(B)** Initial whole-body PET/CT showed hypermetabolic uptake in the right upper-lobe lesion and regional lymph nodes, without definite distant or skeletal metastasis. **(C)** Chest CT during the terminal admission showed post-treatment changes without obvious enlargement of the primary lesion. **(D)** Bone scintigraphy during the terminal admission showed no definite bone metastasis.

A 76-year-old Chinese man with a history of smoking and a baseline Eastern Cooperative Oncology Group performance status (ECOG PS) of 1 was diagnosed with SCLC of the right upper lobe in August 2025. Baseline contrast-enhanced chest CT showed an irregular right upper-lobe mass measuring approximately 35 × 20 mm, with bronchial involvement. Bronchoscopic biopsy of the right upper-lobe lesion confirmed SCLC. PET/CT demonstrated hypermetabolic lymph nodes at the 2R and 4R mediastinal stations and in the right hilar region. Endobronchial ultrasound showed enlarged 4R and 11R lymph nodes, and fine-needle aspiration of the 11R node confirmed metastatic SCLC. No definite distant or skeletal metastasis was identified on PET/CT, and brain MRI showed no intracranial metastasis. The disease was clinically staged as cT2aN2bM0, stage IIIB, according to the ninth edition of the TNM classification and was classified as limited-stage SCLC. Representative baseline and follow-up imaging findings are shown in **Figure 1**.

The patient received six cycles of etoposide plus carboplatin from August 16, 2025, to January 3, 2026. Chest CT after two cycles showed a reduction in the extent of the right upper-lobe lesion and partial regression of the involved hilar and mediastinal lymph nodes. Subsequent imaging showed maintenance of this tumor regression, and the radiological response after completion of chemotherapy was classified as a partial response (PR) according to Response Evaluation Criteria in Solid Tumors version 1.1 (RECIST 1.1). He subsequently received thoracic intensity-modulated radiotherapy to the right lung and mediastinum from January 19 to February 10, 2026, delivered twice daily in 30 fractions to a total dose of 45 Gy. Post-radiotherapy CT examinations on February 24 and March 11 showed treatment-related changes in the right lung without unequivocal tumor progression, and the PR was maintained according to RECIST 1.1. No further antitumor therapy was administered after completion of thoracic radiotherapy. He had a history of right internal jugular venous thrombosis and had been receiving rivaroxaban 10 mg once daily, which was discontinued on May 29, 2026. He did not receive aspirin, clopidogrel, or other antiplatelet therapy.

In May 2026, approximately 3 months after completion of thoracic radiotherapy and more than 4 months after the last cycle of chemotherapy, the patient developed progressive fatigue and dyspnea for 1 week and was admitted on May 25, 2026. At admission, he reported poor appetite and mild cough with a small amount of yellow sputum. No overt hemoptysis, gross hematuria, massive gastrointestinal bleeding, or extensive cutaneous ecchymosis was documented. Physical examination showed no palpable superficial lymphadenopathy or hepatosplenomegaly.

**Figure 2:**
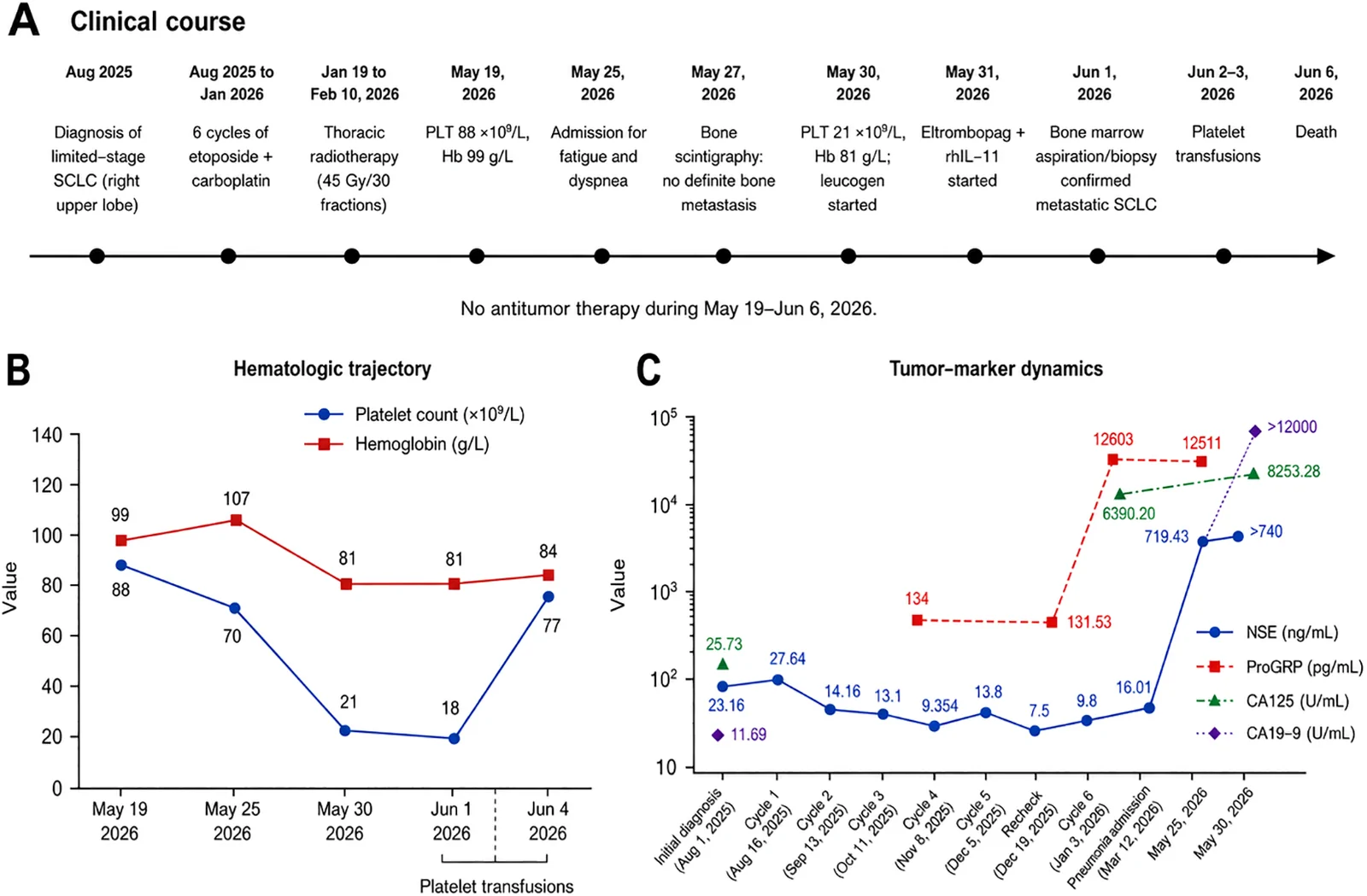
Rapid thrombocytopenia and explosive tumor-marker elevation preceding the diagnosis of bone marrow metastasis. **(A)** Clinical timeline from initial diagnosis to death, including chemotherapy, thoracic radiotherapy, terminal hospitalization, bone marrow examination, platelet transfusion, and outcome. **(B)** Dynamic changes in platelet count and hemoglobin during the terminal hospitalization. **(C)** Dynamic changes in serum tumor markers from the initial treatment course to terminal disease progression.

Laboratory tests revealed rapidly progressive cytopenia. The platelet count decreased from 88×10^9/L on May 19 to 70×10^9/L on May 25, 21×10^9/L on May 30, and 18×10^9/L on June 1. Hemoglobin also decreased to 81 g/L by May 30. Nucleated red blood cells were detected in peripheral blood, and red cell distribution width was increased. D-dimer levels were elevated, but fibrinogen remained mildly elevated rather than depleted. Fecal occult blood was positive, and transient epistaxis occurred on May 29 and stopped spontaneously. The dynamic changes in platelet count and hemoglobin are shown in **Figure 2B**.

Serum tumor markers increased markedly during the final hospitalization. On May 25, neuron-specific enolase (NSE) was 719.34 ng/mL, pro-gastrin-releasing peptide (ProGRP) was 12,603 pg/mL, carbohydrate antigen 125 (CA125) was 6,390.20 U/mL, and carcinoembryonic antigen was 7.82 ng/mL. On May 30, NSE exceeded the upper detection limit (>740 ng/mL), ProGRP remained markedly elevated at 12,511.56 pg/mL, CA125 increased to 8,253.28 U/mL, and carbohydrate antigen 19–9 exceeded 12,000 U/mL. Lactate dehydrogenase and ferritin were also markedly elevated. The overall clinical course and longitudinal tumor-marker changes are summarized in **Figures 2A, C**.

Radiological evaluation during the final hospitalization did not reveal definite skeletal metastasis or obvious enlargement of the primary lung lesion. Chest computed tomography (CT) on May 25 showed post-treatment changes in the right lung and bilateral inflammatory changes. Enhanced abdominal CT showed liver parenchymal abnormalities suggestive of liver dysfunction but no definite intra-abdominal metastatic mass or enlarged abdominal lymph nodes. Bone scintigraphy on May 27 showed no definite bone metastasis. Brain MRI showed no intracranial metastasis. Thus, conventional imaging did not demonstrate unequivocal progression of the previously responsive thoracic disease despite the rapidly worsening cytopenias and marked biochemical progression. These imaging findings are summarized in **Figure 1**.

**Figure 3:**
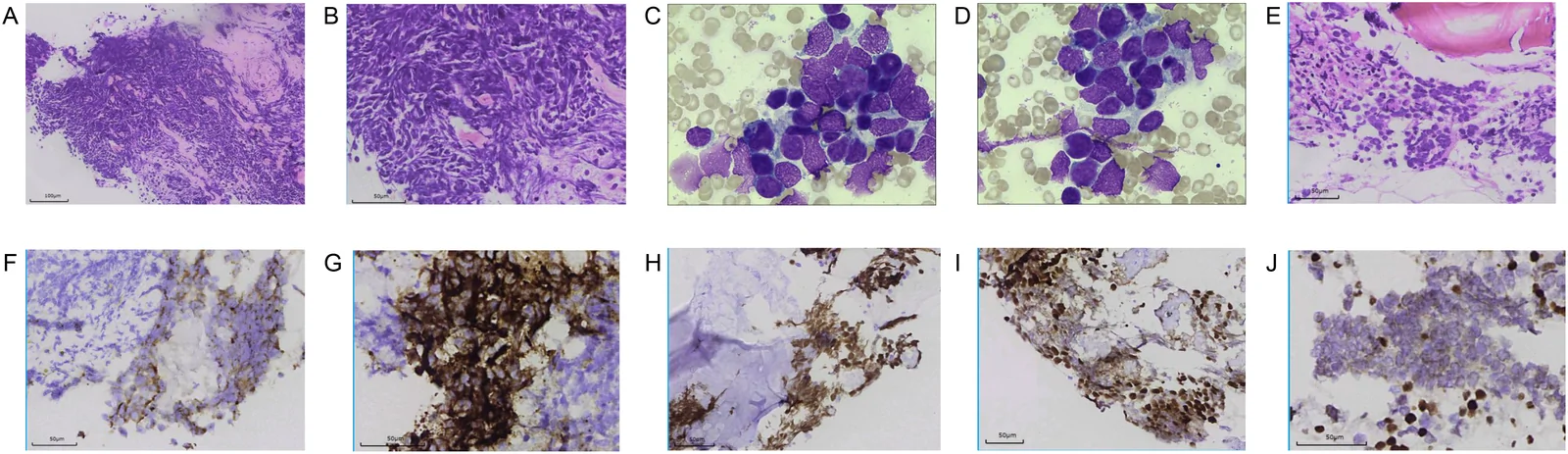
Pathological confirmation of bone marrow metastasis from small cell lung cancer. **(A, B)** Initial bronchoscopic biopsy showing malignant small round cell tumor cells consistent with small cell lung cancer. **(C, D)** Bone marrow smear showing clustered abnormal tumor cells with high nuclear-to-cytoplasmic ratios and hyperchromatic nuclei. **(E)** Bone marrow biopsy showing infiltration by metastatic tumor cells. **(F–H)** Immunohistochemistry showing epithelial and neuroendocrine differentiation, including CKpan positivity, neuroendocrine marker positivity, and TTF-1 positivity. **(I, J)** Immunohistochemistry showing mutant-type p53 expression and loss of RB expression, supporting metastatic small cell lung cancer.

Because the rapidly progressive thrombocytopenia and anemia could not be explained by recent chemotherapy, bone marrow aspiration and biopsy were performed on June 1, 2026. Bone marrow smear showed a prominent population of abnormal cells, accounting for 41.75% of nucleated cells. These cells were heterogeneous in size, with round or irregular nuclei, increased nuclear-to-cytoplasmic ratio, coarse chromatin, and visible clustering. Megakaryocytes were rare, and platelets were reduced in the smear. Conventional karyotyping showed 46, XY, without clonal chromosomal abnormalities. Flow cytometry of the bone marrow detected an abnormal cell population accounting for approximately 5.5% of nucleated cells. These cells were negative for CD45 and expressed CD326, CD56, and CD117, while lacking markers supporting lymphoid or myeloid hematologic malignancy. Bone marrow biopsy confirmed metastatic SCLC. Immunohistochemistry showed positivity for CKpan, epithelial membrane antigen, synaptophysin, chromogranin A, thyroid transcription factor-1, CD56, and INSM1. p53 showed a mutant-type expression pattern, and RB expression was absent. The tumor cells were negative for p40 and p63. Representative bone marrow morphology and immunohistochemical findings are shown in **Figure 3**.

Targeted next-generation sequencing (NGS) was subsequently performed using the residual bone marrow specimen obtained during the same hospitalization. The assay detected clinically relevant or potentially relevant alterations, including RB1 p.Gln689Ter, TP53 p.Val272Leu, KMT2D p.Ser4783Ter, and DNMT3A p.Arg771Ter. The variant allele frequencies of RB1 p.Gln689Ter and TP53 p.Val272Leu were 40.16% and 42.58%, respectively, concordant with the immunohistochemical findings of RB loss and aberrant p53 expression. No actionable common lung cancer driver alteration was detected, and the tumor was microsatellite stable.

The patient received supportive treatment, including discontinuation of rivaroxaban, hemostatic management, leucogen, eltrombopag, recombinant human interleukin-11, and platelet transfusion. Single-donor platelet transfusions were administered on June 2 and June 3, with a transient increase in platelet count to 77×10^9/L on June 4. However, his condition continued to deteriorate. By June 5, he had developed progressive weakness, jaundice, abdominal pain, oliguria, worsening hepatic and renal dysfunction, and clinical features consistent with terminal malignancy and multiorgan failure. On June 6, he developed hypotension, altered consciousness, labored breathing, and bradycardia. At the request of the family, further resuscitative measures were not pursued; the patient was discharged for end-of-life care and died later the same day.

All procedures performed in this case report were in accordance with the ethical standards of the institutional and/or national research committee and with the Declaration of Helsinki and its subsequent amendments. Written informed consent was obtained from the patient’s next of kin for publication of this case report and accompanying images. A copy of the written consent is available for review by the editorial office of this journal.

## 3 Discussion

Previous reports have established that SCLC can involve the bone marrow and cause clinically significant cytopenias (7, 8). The contribution of the present case therefore lies not in documenting marrow involvement itself, but in defining a readily overlooked pattern of relapse. The rapid decline in platelet count and the marked increase in neuroendocrine tumor markers contrasted with negative bone scintigraphy, the absence of destructive skeletal lesions and no obvious enlargement of the treated pulmonary lesion. This biochemical-radiographic discordance suggested that conventional imaging underestimated the extent of disease. Bone marrow morphology and biopsy provided direct evidence of metastatic SCLC, whereas immunohistochemistry and flow cytometry characterized the epithelial and neuroendocrine phenotype. The molecular findings were consistent with SCLC biology but should be regarded as supportive rather than proof of clonal continuity with the original lung tumor. Taken together, these observations show how clinically aggressive marrow relapse may remain radiographically occult until severe hematological deterioration develops. A key lesson from this case is that negative bone imaging does not exclude bone marrow metastasis. Bone scintigraphy is useful for detecting osteoblastic activity or reactive bone turnover, but it may be insensitive when malignant cells predominantly infiltrate the marrow compartment without causing overt cortical destruction or radiographically apparent osseous lesions (14, 15). In the present case, bone scintigraphy did not identify definite skeletal metastasis, and cross-sectional imaging did not show destructive bone lesions. Nevertheless, bone marrow aspiration revealed abundant abnormal cells, and bone marrow biopsy confirmed metastatic SCLC. Therefore, “bone marrow metastasis” is a more accurate description than “bone metastasis” in this patient. This distinction is clinically meaningful because marrow involvement may initially manifest as cytopenia and leukoerythroblastosis rather than bone pain or radiographic bone lesions.

Historical staging series reported bone marrow involvement in approximately 15–30% of patients with newly diagnosed SCLC, whereas isolated marrow involvement occurred in fewer than 5% (6, Ref A). Because confirmed marrow metastasis constitutes distant metastatic disease, modern incidence estimates for each nonmetastatic TNM stage are not available; most evidence derives from older limited-stage/extensive-stage cohorts. Routine bone marrow sampling is not recommended, but aspiration and trephine biopsy should be considered when unexplained cytopenias or nucleated red blood cells suggest marrow infiltration, particularly when imaging is unrevealing (16). In a 95-patient SCLC staging study, the per-patient sensitivity for osseous metastases was 100% for FDG-PET/CT and 37% for bone scintigraphy; however, these estimates should not be extrapolated to isolated marrow-predominant relapse (17). Paired examinations showed similar detection rates for aspiration and trephine biopsy, supporting their complementary use when clinical suspicion remains high (18). Bone marrow involvement indicates extensive disease and short survival, although its independent prognostic effect beyond the overall metastatic burden remains uncertain (19). A retrospective series reported a median survival of 15.7 weeks after the diagnosis of marrow metastasis and an association between chemotherapy and longer survival, but this nonrandomized finding should be interpreted cautiously (19). Treatment follows the principles for recurrent SCLC, but severe cytopenias may restrict the delivery of myelosuppressive therapy and require transfusion and other supportive care. Decisions should therefore be individualized according to performance status, organ function, and hematologic reserve; no marrow-specific systemic regimen has been established.

The differential diagnosis of thrombocytopenia in this patient included prior chemotherapy, drug-related thrombocytopenia, immune thrombocytopenia, bleeding-related platelet consumption, consumptive coagulopathy, and marrow infiltration. The timing of cytopenia argued against acute chemotherapy-induced suppression. Rivaroxaban could increase bleeding risk, but it does not explain severe marrow-replacement thrombocytopenia or the presence of metastatic tumor cells in bone marrow. Although transient epistaxis and fecal occult blood positivity were observed, there was no massive hemorrhage. The elevated D-dimer level suggested malignancy-associated hypercoagulability or a consumptive tendency; however, fibrinogen was not depleted, and the overall pattern was not typical of overt disseminated intravascular coagulation. These findings emphasize that, in relapsed SCLC, severe or rapidly progressive thrombocytopenia should prompt early bone marrow evaluation, particularly when accompanied by anemia, nucleated red blood cells, rising tumor markers, and poor or only transient response to platelet transfusion.

The tumor-marker pattern also provided an important clue. NSE and ProGRP are commonly used neuroendocrine markers in SCLC and may reflect tumor burden and disease activity (20). In this patient, NSE and ProGRP were only mildly elevated or relatively stable during earlier treatment but increased dramatically during the final hospitalization. This discordance between biochemical progression and the absence of obvious radiographic tumor enlargement suggested occult systemic progression. CA125 and CA19–9 were also markedly elevated. Although these markers are not specific for SCLC, they may increase in advanced malignancy, serosal involvement, liver dysfunction, ascites, or systemic inflammatory states. In this case, their sharp increase, together with extremely elevated NSE/ProGRP and pathological confirmation of marrow metastasis, supported widespread biological progression rather than an isolated laboratory abnormality.

Integrated pathological evaluation was central to the diagnosis. Bone marrow morphology showed clustered abnormal non-hematopoietic malignant cells. Flow cytometry detected CD45-negative cells expressing CD326, CD56, and CD117, arguing against a primary hematological malignancy and supporting metastatic epithelial/neuroendocrine tumor cells. Bone marrow biopsy and immunohistochemistry confirmed metastatic SCLC, with positivity for epithelial markers and neuroendocrine markers, including CKpan, EMA, synaptophysin, chromogranin A, CD56, and INSM1, together with TTF-1 positivity. The mutant-type p53 expression pattern and loss of RB were consistent with canonical SCLC biology (13). Contemporary molecular models further emphasize that transcriptional heterogeneity and treatment-associated plasticity can contribute to resistance and relapse in SCLC (21). The rapid marrow relapse after a RECIST 1.1-defined partial response was clinically compatible with aggressive disease evolution, although the present assay did not assess transcriptional subtype or identify a specific resistance mechanism. Targeted NGS identified corresponding TP53 and RB1 alterations that were concordant with the immunohistochemical profile. However, these alterations are not specific to the patient’s original lung tumor and, in the absence of paired sequencing, could not establish clonal relatedness between the marrow lesion and the primary SCLC.

This case also highlights both the value and the limitations of bone marrow-based NGS. The detection of RB1 and TP53 alterations with relatively high variant allele frequencies was consistent with the pathological diagnosis and the immunohistochemical profile. KMT2D alteration may reflect additional epigenetic dysregulation (22, 23), although its clinical significance in this case remains uncertain. By contrast, the DNMT3A p.Arg771Ter alteration should be interpreted cautiously. DNMT3A is a common gene associated with clonal hematopoiesis, and low-variant-allele-frequency DNMT3A alterations detected in bone marrow may originate from hematopoietic clones rather than tumor cells (24, 25). Therefore, molecular findings from tumor-only sequencing of bone marrow specimens should be interpreted in conjunction with morphology, immunohistochemistry, flow cytometry and variant allele frequencies.

This report has several limitations. Paired sequencing of the primary lung tumor and the bone marrow lesion was unavailable; therefore, clonal relatedness between the two sites could not be established. Bone marrow NGS was also performed without a matched normal control, preventing definitive distinction between tumor-derived variants and non-tumor-derived alterations, including those associated with clonal hematopoiesis. Accordingly, the TP53 and RB1 alterations should be interpreted as findings consistent with canonical SCLC biology rather than independent evidence that the marrow lesion was clonally derived from the original lung tumor. In addition, the patient deteriorated rapidly after the diagnosis of bone marrow metastasis and did not receive further antitumor therapy; therefore, the therapeutic implications of the genomic findings could not be evaluated. Nevertheless, this case is instructive because it shows that progressive cytopenia and marked tumor-marker elevation may reveal occult marrow relapse even when conventional imaging does not show definite bone metastasis. Early bone marrow examination should be considered in similar patients with relapsed SCLC.

## 4 Conclusions

Radiographically occult bone marrow metastasis should be considered in patients with relapsed SCLC who develop rapidly progressive thrombocytopenia and anemia, particularly when cytopenia occurs months after chemotherapy and is accompanied by leukoerythroblastosis or marked elevation of neuroendocrine tumor markers. Negative bone scintigraphy or cross-sectional imaging does not exclude marrow involvement. Early bone marrow aspiration and biopsy remain essential for diagnosis in this setting. Bone marrow morphology and biopsy with immunohistochemistry remain central to diagnosis, whereas flow cytometry and genomic profiling provide complementary phenotypic and biological information.

## Data Availability

The original contributions presented in the study are included in the article/**Supplementary Material**. Further inquiries can be directed to the corresponding authors.
