# Supplementary material for: Radiographically occult bone marrow metastasis in relapsed small cell lung cancer presenting with rapidly progressive thrombocytopenia: a case report
Source: Front Oncol. 2026 Aug 26;16:1926902. doi: 10.3389/fonc.2026.1926902 (PMC13553434; doi:10.3389/fonc.2026.1926902)
Supplement: Supplementary file 1 [file Table1.docx]

**CARE Checklist of information to include when writing a case report**

| **Topic** | **Item No** | **Checklist item description** | **Reported on Page Number/Line Number** | **Reported on Section/Paragraph** |
| --- | --- | --- | --- | --- |
| Title | 1 | The diagnosis or intervention of primary focus followed by the words “case report” | Page 1, lines 1–3 | Title |
| Key Words | 2 | 2 to 5 key words that identify diagnoses or interventions in this case report, including "case report" | Page 2, lines 44–48 | Keywords |
| Abstract  (Structured summary) | 3a | Background: state what is known and unknown; why the case report is unique and what it adds to existing literature. | Page 2, lines 20–25 | Abstract/Background |
|  | 3b | Case Description: describe the patient’s demographic details, main symptoms, history, important clinical findings, the main diagnosis, interventions, outcomes and follow-ups. | Page 2, lines 26–42 | Abstract/Case Description |
|  | 3c | Conclusions: summarize the main take-away lesson, clinical impact and potential implications. | Page 2, lines 43–45 | Abstract/Conclusions |
| Introduction | 4 | One or two paragraphs summarizing why this case is unique **(may include references)** | Page 4, lines 78–106 | Introduction, paragraphs 1–3 |
| Patient Information | 5a | De-identified patient specific information | Page 4, lines 107–110 | Case Presentation, paragraph 1 |
|  | 5b | Primary concerns and symptoms of the patient | Page 4, lines 119–123 | Case Presentation, paragraph 3 |
|  | 5c | Medical, family, and psycho-social history including relevant genetic information | Page 4, lines 115–118 | Case Presentation, paragraph 2 |
|  | 5d | Relevant past interventions with outcomes | Page 4, lines 111–118 | Case Presentation, paragraph 2 |
| Clinical Findings | 6 | Describe significant physical examination (PE) and important clinical findings | Page 4, lines 119–130; Page 5, lines 127–132 | Case Presentation, paragraphs 3–4 |
| Timeline | 7 | Historical and current information from this episode of care organized as a timeline | Page 11, lines 330–333 | Figure 2A and legend |
| Diagnostic Assessment | 8a | Diagnostic testing (such as PE, laboratory testing, imaging, surveys). | Page 4, lines 107–118; Page 5, lines 127–164 | Case Presentation, diagnostic paragraphs |
|  | 8b | Diagnostic challenges (such as access to testing, financial, or cultural) | Page 4, lines 88–99; Page 6, lines 186–195 | Introduction paragraph 2; Discussion paragraph 2 |
|  | 8c | Diagnosis (including other diagnoses considered) | Page 5, lines 146–158; Page 6, lines 196–210 | Case Presentation marrow diagnosis; Discussion differential diagnosis |
|  | 8d | Prognosis (such as staging in oncology) where applicable | Page 4, lines 107–114; Page 5, lines 169–173 | Case Presentation, staging and outcome |
| Therapeutic Intervention | 9a | Types of therapeutic intervention (such as pharmacologic, surgical, preventive, self-care) | Page 5, lines 164–170 | Case Presentation, treatment paragraph |
|  | 9b | Administration of therapeutic intervention (such as dosage, strength, duration) | Page 4, lines 111–118; Page 5, lines 164–169 | Case Presentation, treatment details |
|  | 9c | Changes in therapeutic intervention (with rationale) | Page 4, lines 115–118; Page 5, lines 164–170 | Case Presentation, treatment changes |

| Follow-up and Outcomes | 10a | Clinician and patient-assessed outcomes (if available) | Page 5, lines 169–173 | Case Presentation, outcome paragraph |
| --- | --- | --- | --- | --- |
|  | 10b | Important follow-up diagnostic and other test results | Page 5, lines 146–164; Page 5, lines 167–170 | Case Presentation, marrow and follow-up test results |
|  | 10c | Intervention adherence and tolerability (How was this assessed?) | Page 5, lines 164–170 | Case Presentation, supportive treatment paragraph |
|  | 10d | Adverse and unanticipated events | Page 5, lines 170–173 | Case Presentation, clinical deterioration and death |
| Discussion | 11a | A scientific discussion of the strengths AND limitations associated with this case report | Page 7, lines 239–247 | Discussion, limitations paragraph |
|  | 11b | Discussion of the relevant medical literature **with references** | Page 6, lines 178–225; Page 7, lines 226–238 | Discussion, literature-based interpretation |
|  | 11c | The scientific rationale for any conclusions (including assessment of possible causes) | Page 6, lines 178–210; Page 7, lines 219–238 | Discussion, causal interpretation and NGS interpretation |
|  | 11d | The primary “take-away” lessons of this case report (without references) in a one paragraph conclusion | Page 7, lines 248–257 | Conclusions |
| Patient Perspective | 12 | The patient should share their perspective in one to two paragraphs on the treatment(s) they received | Not available | Not included |
| Informed Consent | 13 | Did the patient give informed consent? Please provide if requested | Yes; page 8, lines 269-274 | Footnote |

Please leave this space alone as it will be supplemented by the editorial office when needed.
